# Supplementary material for: Patterns of Intron Gain and Loss in Fungi
Source: PLoS Biol. 2004 Nov 30;2(12):e422. doi: 10.1371/journal.pbio.0020422 (PMC532390; doi:10.1371/journal.pbio.0020422)
Supplement: Table S1 — Also available at http://genes.mit.edu/NielsenEtAl/. (4.3 MB ZIP). [file pbio.0020422.st001.zip › NielsenEtAl/html/570.html]

AN4602.1.NCU02264.1.MG00904.1.FG06294.1


```
 CLUSTAL W (1.82) Multiple Sequence Alignments - Introns Inserted


Sequence 1: NCU02264.1	204 aa
Sequence 2: MG00904.1	211 aa
Sequence 3: FG06294.1	201 aa
Sequence 4: AN4602.1	193 aa
Alignment Length: 215 aa
Number Identitical Residues: 105 aa
Alignment Score (without introns) 4888


MG00904.1 	MSEKGKAVASA~AG2DDTTTPSNPRGIPKAPFVDNVEDYVTSRAEVESTLVRFQEMIS2K
NCU02264.1	MATKGKEVETA~GR2D--ATPANPRGIPQAPFVDKVEDYVTSRADVEPTLRRFQELIA~K
FG06294.1 	MASKGKEAATS2KD~A---TPTNPRGIPYAPFVDKVEDYVTTRDDVEPTLRSFQEMIS2K
AN4602.1  	MADK-----KP~SD2P---TQTNPRGIPVAPFVDNVSDYVSSRADVEPTMRSFQEMIS~K
          	*: *     ..        * :****** *****:*.***::* :**.*:  ***:*: *

MG00904.1 	YQFMELNLQRRVAGLKGKLPDIEKTLETVQFLKLSK~DGG--DPIETSFELNDTLYTKAE
NCU02264.1	YQFMEQNLQRRVVGLKDKMPDIQKTLDTVRFLQMRK0DES--DPIETTFELNETLYAQAK
FG06294.1 	YQFMEMNLQKRMGGLKEKIPDIQKTLDSVKFLKLRK0DDD--EAIETTFELNDTLYSKAK
AN4602.1  	YQFMEVNTSRRAAGLREKIPDIKKTLEMVTFLKARK~ESSSATPLETNFELNDTLYARAT
          	***** * .:*  **: *:***:***: * **:  * : .:: .:**.****:***::* 

MG00904.1 	IPP--TDEVFLWLG0ANVMLSYPIDEAEELLDSKQKAAKQSLQNCEEDLEFLREQIT0TM
NCU02264.1	IPP--TDEVYIWLG~ANVMLSYPIEEAEQLLDSKLKAATQSLQNCEEDLDFLREQIT0TM
FG06294.1 	IPA--TEEVYIWLG0ANVMLSYPIDEAETLLSSKLSTAKTSLSNCEEDLDFLREQIT0TM
AN4602.1  	VDPAHTEEVYLWLG~ANVMLAYPIAEAQAMLEDKLAAAEQSLANCDEDLEFLREQIT0TL
          	: .: *:**::*** *****:*** **: :*..*  :*  ** **:***:******* *:

MG00904.1 	EVALARVYNWDVMQKRKEKAEEDKLKGKDKEKETSKPDNDS
NCU02264.1	EVAVARVYNWDVVQKRKEKEEEEKTKGKKSETASDG-----
FG06294.1 	EVAVARVYNWEVVQKRKDKAVEEEENKKVKSQGD-------
AN4602.1  	EVATARVYNWDVVQRRKERAE-----GKEETS---------
          	*** ******:*:*:**::        * .
```
